# Comprehensive processing of high-throughput small RNA sequencing data including quality checking, normalization, and differential expression analysis using the UEA sRNA Workbench

**DOI:** 10.1261/rna.059360.116

**Published:** 2017-06

**Authors:** Matthew Beckers, Irina Mohorianu, Matthew Stocks, Christopher Applegate, Tamas Dalmay, Vincent Moulton

**Affiliations:** 1 School of Computing Sciences, University of East Anglia, Norwich, NR4 7TJ, United Kingdom; 2 School of Biological Sciences, University of East Anglia, Norwich, NR4 7TJ, United Kingdom

**Keywords:** high-throughput sequencing (HTS), microRNA (miRNA), small RNA (sRNA), UEA sRNA Workbench, quality checking, normalization, differential expression

## Abstract

Recently, high-throughput sequencing (HTS) has revealed compelling details about the small RNA (sRNA) population in eukaryotes. These 20 to 25 nt noncoding RNAs can influence gene expression by acting as guides for the sequence-specific regulatory mechanism known as RNA silencing. The increase in sequencing depth and number of samples per project enables a better understanding of the role sRNAs play by facilitating the study of expression patterns. However, the intricacy of the biological hypotheses coupled with a lack of appropriate tools often leads to inadequate mining of the available data and thus, an incomplete description of the biological mechanisms involved. To enable a comprehensive study of differential expression in sRNA data sets, we present a new interactive pipeline that guides researchers through the various stages of data preprocessing and analysis. This includes various tools, some of which we specifically developed for sRNA analysis, for quality checking and normalization of sRNA samples as well as tools for the detection of differentially expressed sRNAs and identification of the resulting expression patterns. The pipeline is available within the UEA sRNA Workbench, a user-friendly software package for the processing of sRNA data sets. We demonstrate the use of the pipeline on a *H. sapiens* data set; additional examples on a *B. terrestris* data set and on an *A. thaliana* data set are described in the Supplemental Information. A comparison with existing approaches is also included, which exemplifies some of the issues that need to be addressed for sRNA analysis and how the new pipeline may be used to do this.

## INTRODUCTION

RNA silencing is known to play a key role in the fine-tuning of gene expression in eukaryotes (Brodersen and Voinnet 2006).The process is mediated by a set of RNA molecules referred to as small RNAs (sRNAs). Well-known examples of sRNAs include microRNAs (miRNAs) (Bartel 2009; Voinnet 2009) and small interfering RNAs (siRNAs) (Carthew and Sontheimer 2009; Meister 2013). These RNA fragments are excised by Dicer/Dicer-like proteins from double-stranded RNA precursors deriving either from single stranded RNAs with a hairpin-like secondary structure, the miRNAs (Zhu et al. 2013), or long double-stranded RNA created by a polymerase, the siRNAs (Chen 2012). The sRNAs target and subsequently silence genes and thus play an important role in gene regulation (Lippman and Martienssen 2004; Omidvar et al. 2015), defense against pathogens (Szittya et al. 2010; Donaszi-Ivanov et al. 2013) and general maintenance of the genome (Molnar et al. 2007; Mohorianu et al. 2011).

For most molecular biology experiments, an important question is how the observed phenotype or inherent differences (e.g., time or organ/tissue series) are reflected in the variation in expression of sRNAs, commonly referred to as differential expression analysis or DE analysis (Mohorianu and Moulton 2010; Garber et al. 2011; Ozsolak and Milos 2011; Xu et al. 2014). Identification of DE sequences consists of several distinct stages: first, the quality of the data is investigated to identify (and potentially exclude) samples containing artifacts such as overrepresenting biases originating from sequencing inaccuracies (Sorefan et al. 2012; Raabe et al. 2014) or introduced from the handling of the original biological sample. Second, the reads are annotated to determine which categories of sRNAs are present. Finally, the expression levels in the samples are normalized to improve the comparability between samples and, subsequently, to refine the accuracy of DE predictions (McCormick et al. 2011; Dillies et al. 2013).

Bioinformatics methods developed for DE analysis have thus far largely focused on analyzing messenger RNA (mRNA) data, first from microarray experiments and now, more commonly, from RNA-seq (mRNA-seq) data sets (Rapaport et al. 2013; Soneson and Delorenzi 2013). Many of these approaches devised for each stage of a DE analysis are transferable to sRNA data sets (see Table 1). However, there are a number of conceptual differences between sRNA microarrays, which capture a small number of known sequences (mainly miRNAs), and sRNA-seq, which capture a wider variety of known and novel sRNAs (usually, in excess of 100k unique reads). Similar differences in a number of quantified transcripts are also observed between the output of mRNA-seq experiments and sRNA-seq output. More specifically, for mRNA studies, the expression levels of the reads are aggregated into a gene abundance (Mortazavi et al. 2008), whereas each sRNA sequence contributes individually to the distribution of abundances (McCormick et al. 2011; Studholme 2012). Because of this, the resulting distributions are different both in shape; mRNA-seq abundances have a Gaussian-like distribution whereas sRNA-seq abundances follow an exponential-like distribution, and in number of points; thousands of genes compared to millions of unique sRNAs (Barquist and Vogel 2015). In addition, sRNA-seq data have a higher ratio of noise (random degradation products) to signal (genuine sRNAs); due to the nature of sRNA-seq processing the median of sRNA abundances lies within the noise range (Vidal et al. 2013). This implies that existing methodologies for microarrays or mRNA-seq DE analyses are applicable but not always appropriate for sRNA-seq data sets (McCormick et al. 2011; Gupta et al. 2012; Lohse et al. 2012; Vidal et al. 2013). Therefore, it is important to develop tools that address the specific characteristics of sRNA-seq data sets and their analysis to complement those currently used for mRNA-seq analysis.

**TABLE 1.**
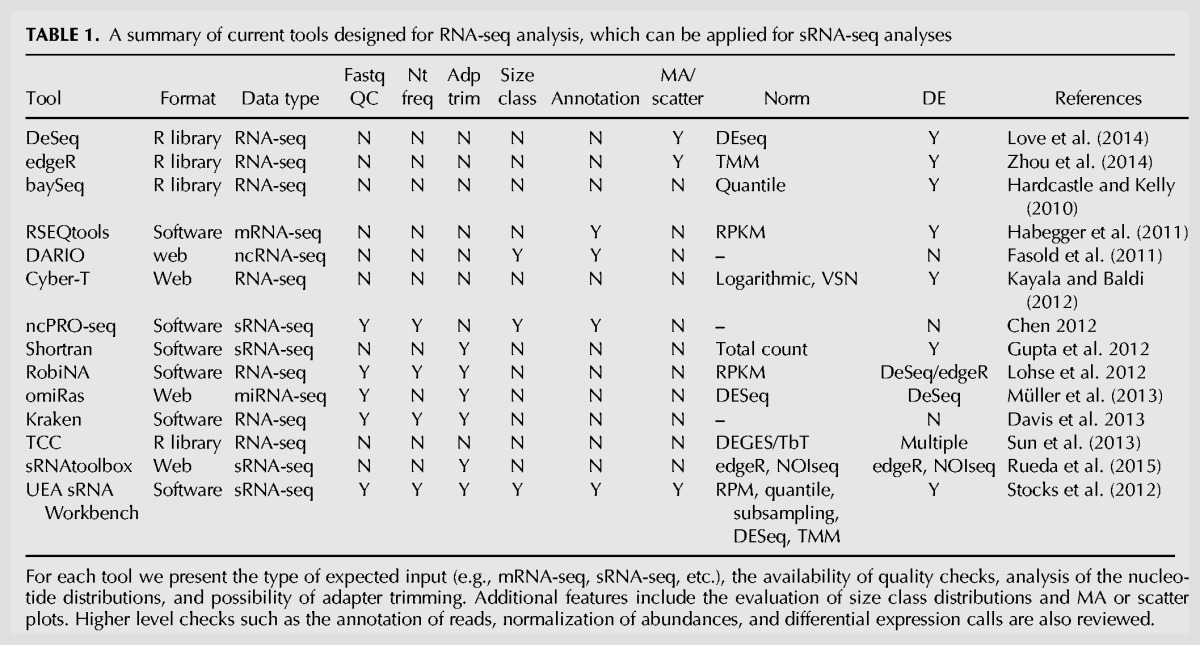
A summary of current tools designed for RNA-seq analysis, which can be applied for sRNA-seq analyses

A common approach for HTS data analysis is to group several tools into a pipeline. As well as providing the ability to tailor pipelines to individual experiments, this enables researchers to configure the distinct stages of the analysis as required (Davis et al. 2013). After the setup is complete the (likely lengthy) procedure can be executed without the need for further input from the user. Currently there are several mRNA-seq pipelines available, such as DESeq/DESeq2 (Anders et al. 2013; Love et al. 2014) or edgeR (Zhou et al. 2014) that can be configured to handle, to some extent, the various stages of a sRNA DE pipeline as well (see Table 1). However, none of these cover the entire analysis of an sRNA data set.

Here we present a comprehensive, interactive processing pipeline for the analysis of sRNA-seq data sets included as part of the UEA small RNA Workbench (Moxon et al. 2008; Stocks et al. 2012). The pipeline summarizes approaches for quality checking (Mohorianu et al. 2011; Axtell 2013), normalization (Dillies et al. 2013), and identification of expression-derived patterns (Lopez-Gomollon et al. 2012; Mohorianu et al. 2013). To enable the user to compare sRNA-seq libraries and indicate the level of confidence to place on predictions made during downstream analysis, we also provide a series of diagnostic plots used throughout the pipeline to assess the characteristics and overall quality of the samples. Users can also evaluate different normalization methods in order to decide which approach is suitable for their data set. In addition, we present a confidence interval (CI)-based approach (Lopez-Gomollon et al. 2012) to summarize the magnitude and direction of fold changes, for each sRNA. On an *H. sapiens* data set, described in the main text, we demonstrate how this can be extended to multiple comparisons that can be used to group sequences with similar patterns across the whole experiment.

## RESULTS

In this section, we illustrate the features of our pipeline on a publically available data set in *H. sapiens*, GSE47532 (Barrett et al. 2013; Camps et al. 2014) to highlight its use to identify characteristics and diagnose problems in real data. Additional examples are presented in Supplemental Information 1 (example on a *B. terrestris* data set) and in Supplemental Information 3 (example on an *A. thaliana* data set). The impact of the number of samples or available memory (RAM) on the runtime is discussed in Supplemental Information 2.

### Workflows and implementation details

**FIGURE 1.**
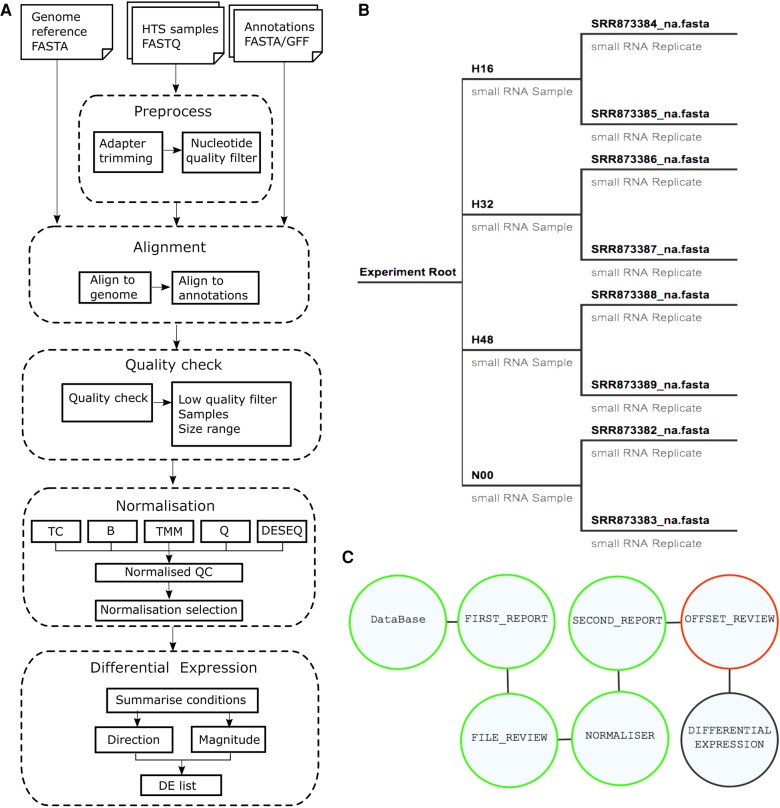
Overview of the analysis pipeline and the input of the Differential expression workflow implemented in the UEA sRNA Workbench. (*A*) Diagram showing the steps of the pipeline, including the preprocessing, alignment to the reference genome and available annotations, quality checking of the raw and processed data, normalization and differential expression call. (*B*) Hierarchical representation of the input data obtained using the input wizard. (*C*) The user interface for a workflow containing Quality Checks, Normalization and Differential Expression call; each node can be configured individually.

The pipeline is part of the existing UEA small RNA Workbench package (Stocks et al. 2012), which provides a user friendly environment designed for all users regardless of computing experience. The latest version of the workbench also facilitates the chaining together of multiple tools within a workflow. This allows each distinct part of a pipeline to be fully configured prior to runtime forgoing the need for many separate programs that require interlinked inputs/outputs. For example, given a set of sRNA samples, a workflow for the identification of DE sRNAs could consist of the quality checking of the samples, the normalization of expression levels, the identification of differentially expressed, annotated reads and the overview of resulting expression patterns—a diagram illustrating this series of steps is presented in Figure 1A. Within the workbench interface, the workflow (Fig. 1C) consists of multiple user configurable nodes that represent the various stages in the analysis.

A standard pipeline takes as input sequence data in FASTA format with the adapters trimmed. The files can be generated using the adapter removal tool (Stocks et al. 2012) which also allows users to process samples created using the HD sequencing protocol (Sorefan et al. 2012). The next step is the configuration of the workflow using the setup wizard. The first stage is to organize the data/samples in a manner that reflects the original wet lab experimental design. The sample hierarchy is represented as a tree diagram where leaf nodes represent the replicates and the parents represent the individual samples (Fig. 1B). Users then provide a reference genome and an (optional) GFF file, corresponding to the genome build, which will be used for the annotation stage. If an annotation file is provided, users can then choose which annotations are relevant for the analysis.

After configuring the sample files, users can choose to begin the workflow immediately or enter each stage of the workflow and change the configurable parameters, as necessary. In addition, during the workflow, users can mark problematic replicates (resulting from the first stage of quality checking) or individual size classes for removal, then select up to six normalization methods to be investigated. The quality check reports are then recreated on the normalized data and can be inspected. Next, the user can select the method that best corrects the data artifacts based on the nuanced characteristics of the data set's expression distributions.

The quality check, normalization, and DE steps are computationally intensive and pose significant demands on both processor and in particular memory (RAM). To counteract this issue, we developed a series of back end improvements, which enable users with a wide range of computing hardware to use the pipeline. More specifically, we used disk solutions based on relational database management interfaced with a Java front end and interacted via a JavaScript GUI (which is also used to display resulting graphs and tabular results). However, as the use of disk for runtime storage and calculations can have significant impacts on processing time, a RAM-only version of the software is also available for users with access to high-end computing hardware.

### Quality checking

**FIGURE 2.**
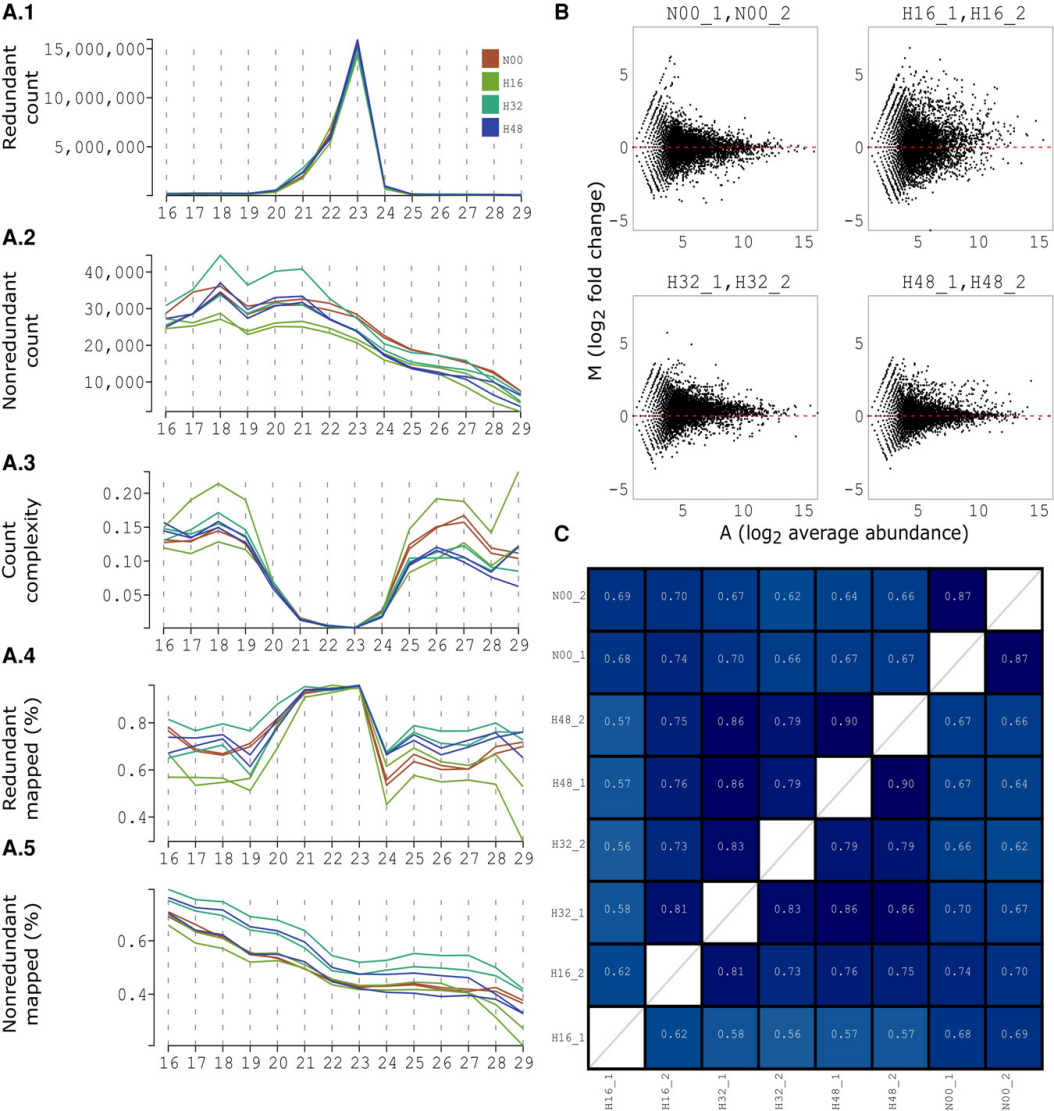
Quality checks for the H data set. (*A*) The characterization of reads within a sample can be obtained by creating the size class distributions for redundant (*A*.1) and non-redundant (*A*.2) reads. Next, the ratio of unique to total reads can be investigated using the complexity distribution (*A*.3). Lastly, the proportions of genome matching reads for redundant (*A*.4) and non-redundant (*A*.5) reads highlight the quality of the sRNA library. (*B*) MA plots on the raw abundances (prior to any normalization or filtering) for evaluating the reproducibility of the replicates. On the *x*-axis we represent the average abundance between the replicates; on the *y*-axis we represent the fold changes. Good samples show low variability with the increase of abundance (e.g., N00, H32, and H48); problematic samples are characterized by high variability between replicates (e.g., H16). (*C*) Jaccard similarity indexes computed on the top 1000 most abundant reads. These indicate a high reproducibility between the N00, H32, and H48 replicates (in excess of 0.8) and a low reproducibility for the H16 replicates (0.62). Interestingly, the second H16 replicate is more similar to the first replicate in the H32 time point.

To illustrate the quality check stage of the pipeline, initial checks on a *H. sapiens* data set (H data) were conducted both before and after aligning reads to the reference genome. The first step of the pipeline is to evaluate the overall features of the data being analyzed. The sequencing quality of individual sRNA-seq samples is initially assessed based on the positional nucleotide composition. Next, the total library size (redundant count) and the total number of unique sequences (non-redundant) count are compared across libraries to assess the variation in sequencing depth. The size class distributions for both redundant and non-redundant reads (Fig. 2A1,A2) can indicate abundant or otherwise important sRNA classes early on in the analysis, or identify issues with the sequencing or mapping of certain size classes. The distribution of complexities, defined as the ratio of redundant to non-redundant reads, provides an approximation of the number and abundances of reads in each size class (Fig. 2A3). Complexity values that are close to 1 indicate a highly diverse set of low abundant sequences, whereas lower complexity values are caused by a smaller set of highly abundant sequences (Mohorianu et al. 2011). For the H data set, we observe a peak in the redundant count distribution at 22–23 nt and a sharp and focused decrease in complexity (Figure 2A1,A3). This indicates the presence of a few highly abundant sRNAs for these particular lengths. We also notice that one replicate of the H32 condition contains more unique reads than the other samples for sizes lower than 22 nt, and that there is a markedly higher complexity for an H16 replicate across the lower and higher range of size classes, indicating an over representation of read variants.

The qualitative replication analysis is conducted through the replicate versus replicate scatter plots and MA plots/Bland–Altman plots (Bland and Altman 1986), Figure 2B, with similar characteristics and interpretation to those on microarray data (Bolstad et al. 2003; McCormick et al. 2011; Dillies et al. 2013); for the latter each dot corresponds to a gene, in this context each dot represents an sRNA. This comparative analysis can be extended to higher levels (such as at the sample or treatment level) and it should be reviewed again using the normalized expression levels. For the H data set, this analysis indicated a high consistency for the H32 and H48 replicates and reduced agreement between the H16 replicates. Supporting the initial observation, the most dispersed size-separated fold changes are those found between the replicates of H16 (Fig. 2B). Low Jaccard indices generated in the second report indicate that these replicates have poor comparability caused by large differences in both the sequence count distribution and sequence composition of the first replicate (Fig. 2C). Since there are only two replicates per treatment and there is no objective approach for choosing one of the two, this plot indicates that the H16 treatment should probably be excluded from further analysis. The other treatments show a high similarity between replicates, with very few fold changes greater than an absolute log_2_ fold change of one at higher average expression levels. Although treatment H32 shows a slight skew toward positive fold changes caused by a higher sequencing depth in the second replicate, the pipeline can be used to correct this issue at the normalization stage.

The percentage of genome-matching reads is also calculated for both redundant and non-redundant sequences and across size classes (Fig. 2A). In addition to examining the entire sRNA population in a data set, all quality checks described so far can also be calculated and compared visually across individual annotations of interest. These include miRNAs, other ncRNAs (such as tRNAs, rRNAs, or snoRNAs), protein-coding genes and repeat/ transposable elements depending on available annotation information (Mortazavi et al. 2008; Xu et al. 2014). These analyses indicate a high proportion of reads in these samples are likely to be miRNAs.

### Normalization

The next step in the pipeline is the normalization of the expression levels. In the normalization node we incorporate several existing methods for normalization, with additional features that we have developed especially for sRNA data sets. For scaling-based methods, the normalization total influences the subsequent DE call; ideally it should not be much lower than the original number of reads. For example, if the scaling is done at 1 M for samples with >10 M reads then all the expression levels will be reduced and DE may be hidden. Alternatively, if scaling is done at 10 M for samples with <1 M reads, then DE could be artificially be generated. An appropriate normalization total therefore lies in the same range as the sample totals (the average and median options are presented as alternatives). Other options are rank-based quantile normalization adapted for sRNA-seq data (Bolstad et al. 2003) and subsampling normalization (Li et al. 2012).

**FIGURE 3.**
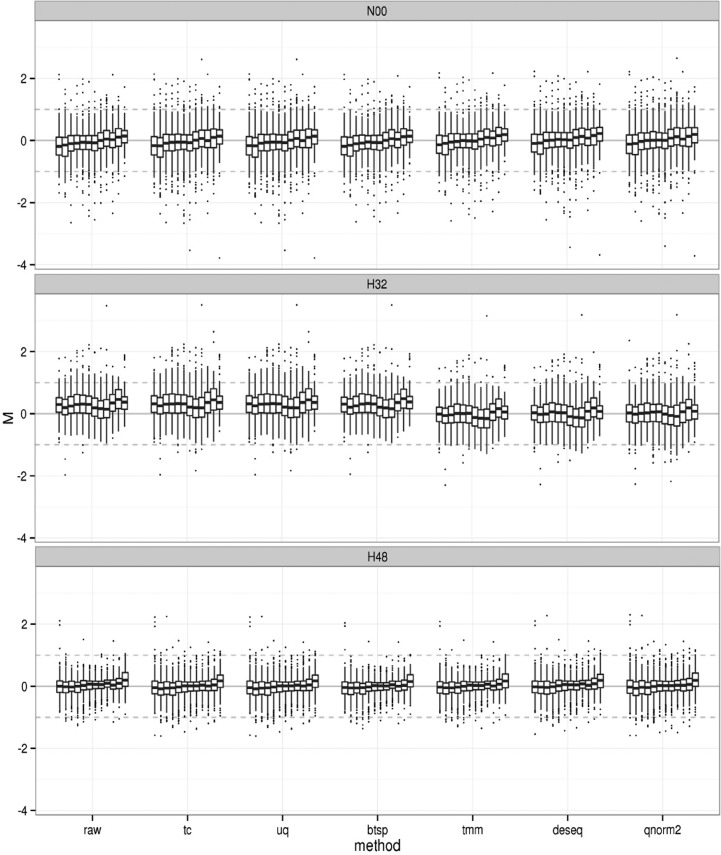
Evaluation of the appropriateness of the normalization methods on the H data set. For each sample and for each set of replicates, we represent the fold-change distributions (*y*-axis) for each individual size class (*x*-axis). Based on the assumption that no significant differences are expected between replicates, a suitable normalization is one that brings all distributions on the 0 line (in log_2_ scale, this corresponds to equal values in both replicates). For the H data set, the TMM, DeSeq2, and adapted quantile normalization fulfill this criterion for all samples.

The analysis of the H data set highlights a common issue with normalization where two replicates are sequenced with different overall depths (mainly due to the characteristics of the sequencing platform used). To evaluate which method(s) are suitable for this data set, we tested all six normalization techniques described in Materials and Methods. Figure 3 illustrates the size-separated distributions of differential expression which can be used to evaluate the suitability of each normalization method. Fold-changes between replicates should be minimal and produce a distribution centered on zero, after normalization. While the TMM, DESeq2, and quantile methods appear to center the distributions of all size classes, the total count, subsampling, and upper quartile methods do not improve on the comparability of the distributions. This suggests that for the H data, either TMM, DESeq, or quantile normalization should be chosen as normalization approaches.

### Differential expression comparison with existing approaches

We exemplify the DE analysis on the H data set for two comparisons: N00/H32 and H32/H48; the left-hand side is considered the reference sample.

**FIGURE 4.**
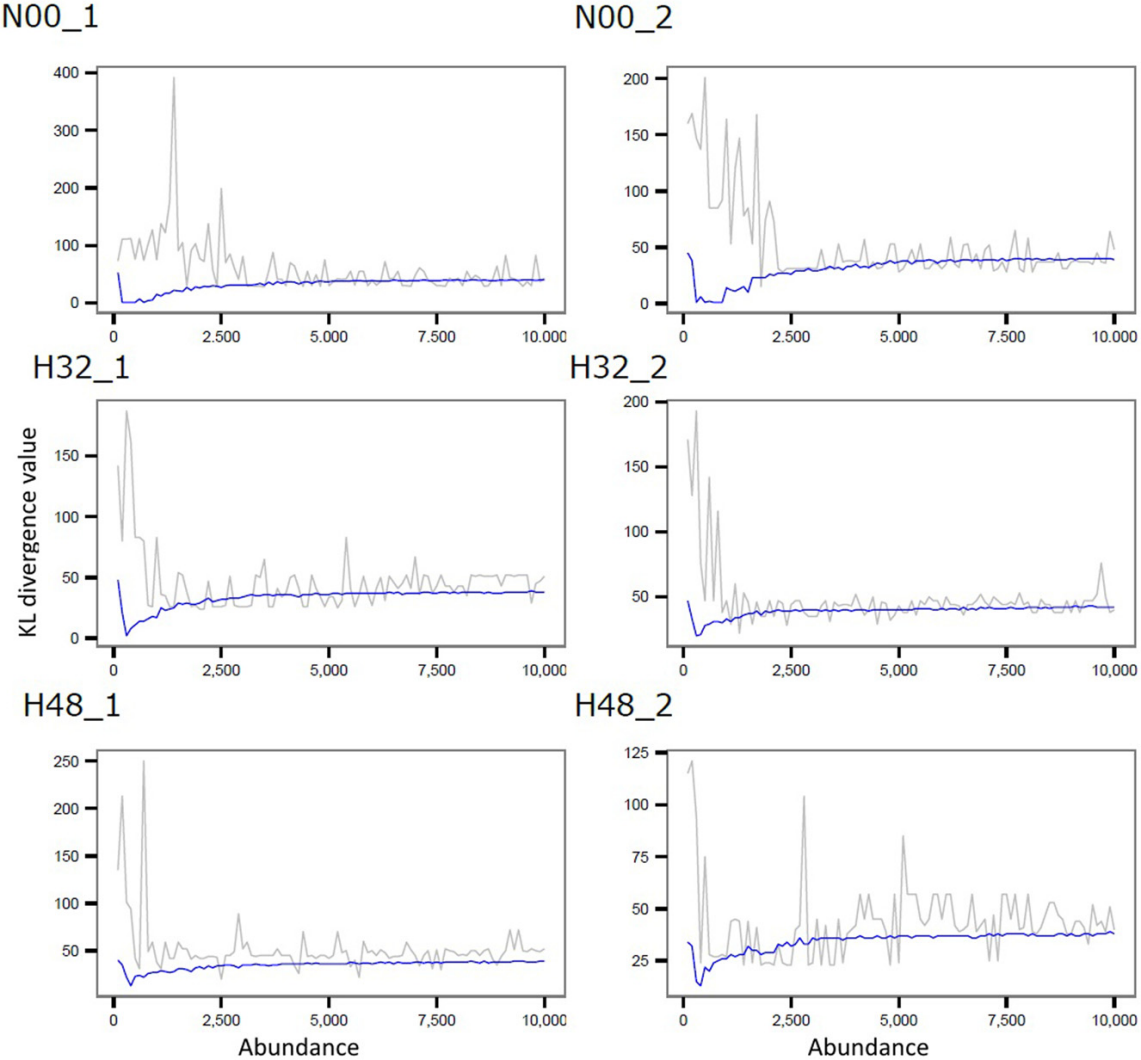
Identification of an offset for each sample in the H data set using Kullback–Leibler divergence to compare the strand bias distributions of reads to a random uniform distribution, in windows of various lengths. This analysis was done on windows of length 1000 nt (parameter that can be modified from the GUI), for each of the two replicates (_1 and _2) of the three accepted samples, N00, H32, and H48. On the *x*-axis we show the abundances of the considered windows (the abundance within a window is the algebraic sum of the abundances of all incident reads); on the *y*-axis we represent the value of the KL divergence. The gray line indicates the unsmoothed KL divergence values and the blue line shows the divergence values smoothed by LOESS (span = 0.3). The offset for each sample is determined as the minimum of the smoothed divergence. The offset for the whole data set is the overall minimum of these values; for this data set this value was determined to be 42.

We compared our results, obtained using the offset fold change, in log_2_ scale (LOFC) and confidence interval (CI) pattern approach—described in Materials and Methods, with two of the most widely used tools for detecting DE reads, DESeq2 (Love et al. 2014), and edgeR (Zhou et al. 2014). Both approaches control for false positives by estimating dispersions and weighting fold changes based on these dispersion estimates. For the DESeq2 and edgeR analyses we used a significance cut-off of 0.05. For the method implemented in the workbench, we applied a threshold of 1 LOFC (both for U and D patterns) to call sequences as DE. This was selected based on empirical evidence that a sequence with a log_2_ fold change of one can be detectable on a Northern blot or via qPCR (Morey et al. 2006). The KL divergence curves generated from the H data set used for determining the appropriate offsets are shown in Figure 4. We also assessed the dependence of the offset on the number of strand bias bins and length of the alignment window. In the H data set, the number of strand bias bins heavily affected the resulting offset up to 100 bins, after which point the KL curve remains unchanged, which resulted in an offset biased toward the lower end of abundance levels. The offset was also affected by alignment window length and can vary erratically when using the raw measures; however, we utilize a LOESS smoothing function (Cleaveland 1979) to produce a more stable offset.

**FIGURE 5.**
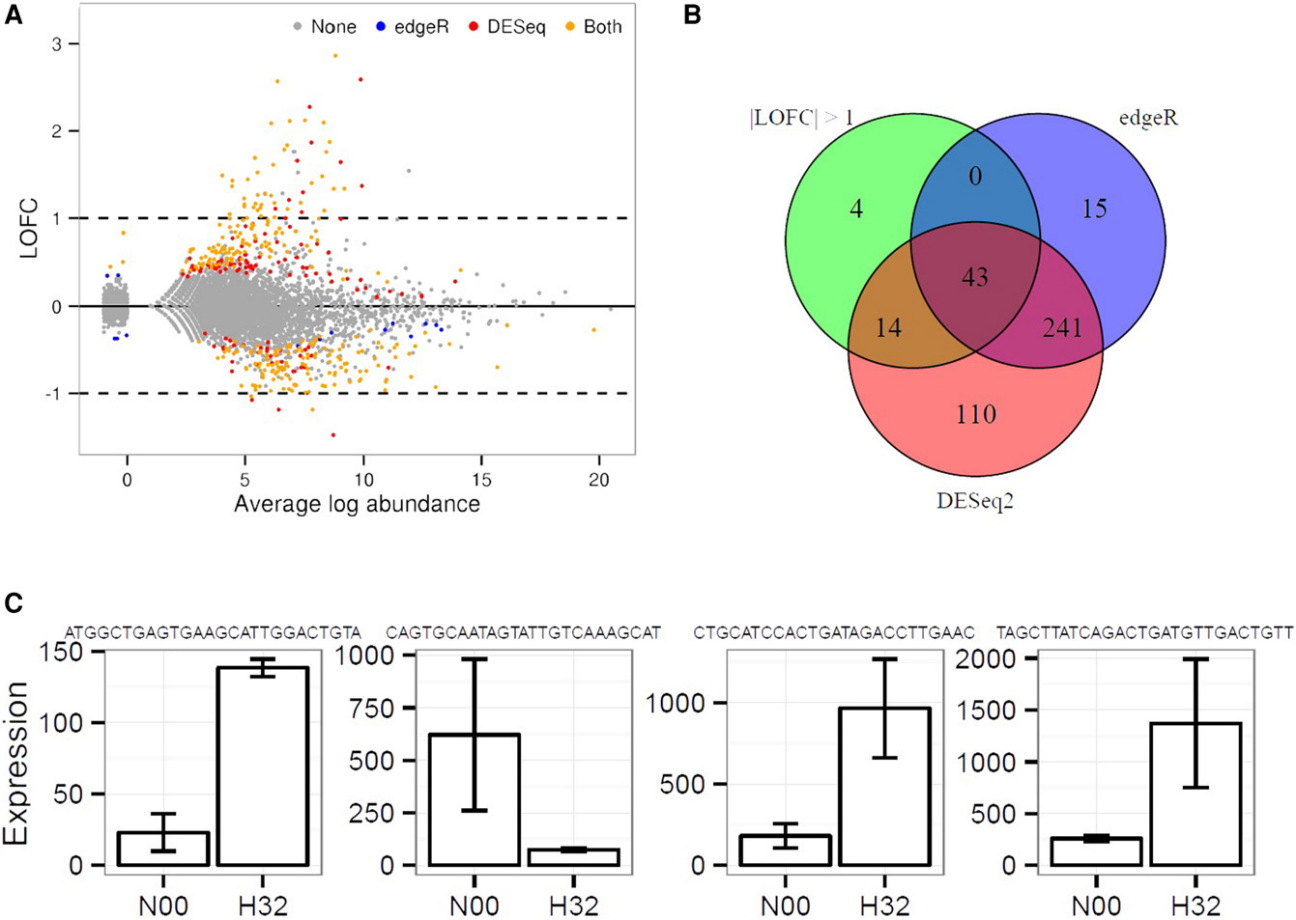
Assessment of three approaches used for the identification of differentially expressed reads applied on the N00 versus H32 comparison (H data set). (*A*) MA plot created using the normalized expression levels (TMM method, see Fig. 3). On the *x*-axis we represent the average abundance; on the *y*-axis we represent the log_2_(OFC). The color of the dots indicates whether the reads were called DE by both edgeR and DESeq2 (orange), exclusively by edgeR (blue), or exclusively by DESeq2 (red). Reads accepted as DE using the LOFC approach are those *outside* the dotted lines. (*B*) Venn diagram showing the number of reads called DE using the LOFC, edgeR, and DESeq2 methods. (*C*) Distributions of expression levels (represented as maximal intervals) for the four sequences called DE exclusively by the LOFC method.

For the N00 versus H32 comparison of the H data set, 427 sequences were called DE by all methods (Fig. 5B). DESeq2 and edgeR both predicted 241 sequences not called DE by the LOFC method; DESeq2 returned 110 differentially expressed sequences that edgeR and our method did not find significant, and edgeR predicted 15 differentially expressed sequences which were not captured using the other methods. Based on the MA plot (Fig. 5A) we observe that the abundance and/or offset fold-change of these specific calls is low. These artifacts can be identified and evaluated on a case-by-case basis by using the LOFC and the CI approach. In addition, we present the expression levels of the four reads identified exclusively using the LOFC approach (Fig. 5C).

## DISCUSSION

We have described a sRNA processing pipeline, part of the UEA sRNA Workbench, that includes steps for quality checking, normalization, and identification of DE sRNAs considering the unique characteristics of sRNA-seq data sets. To achieve a better understanding of these data sets, the pipeline generates a set of diagnostic plots, which can be used initially to review the raw sequencing quality of the replicates and then to assess the effect that different normalization techniques have of the abundance distributions. The use of a suitable normalization is essential for reducing false-positive predictions; however, no single normalization technique can be invariably applied to all sRNA-seq data sets. To evaluate which approach is appropriate for a given data set (i.e., by rendering the samples comparable from most [preferably all] quality check angles) we encourage the user to investigate their using the revised quality check plots.

When identifying DE transcripts in HTS data it is important to take into account the level of noise, a quantity that increases with the depth of sequencing. To account for this, we have implemented a user-friendly tool for the identification of a suitable offset, which estimates the abundance range of the reads lacking sRNA characteristics (e.g., specific size), taking into account the sequencing depth and the characteristics of the sRNA population present in the samples. We compared the results of our DE analysis (LOFC) to that of DESeq2 and edgeR DE packages to determine the level of overlap between other methods and our own. In lieu of a *P*-value threshold to assess DE genes, which often reports large numbers of significant genes often with a low difference in expression, we used a cut-off of 1 LOFC to filter the reported sequences. The cut-off can, however, be user defined in order to reduce or increase the number of reported sequences. Importantly, the ranking of sequences by LOFC is not populated with high but insignificant fold changes.

**FIGURE 6.**
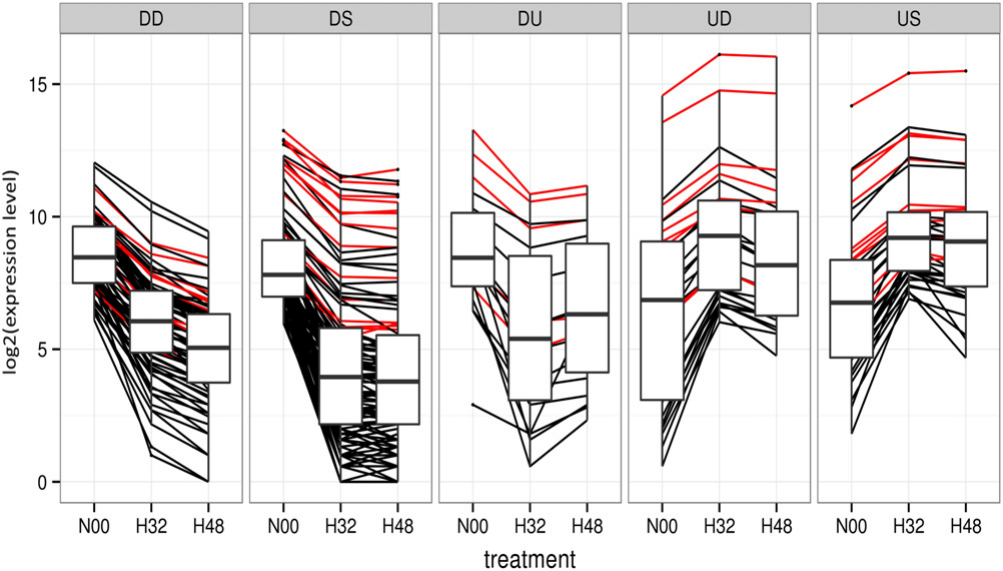
Clusters of reads sharing similar patterns (only the clusters with more than 15 entries were presented; the SS cluster was excluded, since the vast majority of the reads are not expected to be differentially expressed between treatments). The U and D descriptors were assigned to reads for which the LOFC on the proximal ends of the maximal expression intervals was in excess of one. Each line corresponds to the averaged expression profile, on the two available replicates, for one sRNA; the red lines are used to highlight miRNA expression profiles. The boxplot interquartile ranges (IQRs) are used to highlight the distributions of expression in each time point and *underline* the pattern.

To further accommodate the variability between replicates we use CIs created over normalized replicate expression levels which produce more stringent lists of DE sequences between treatments. The method is also extended to multiple conditions by using pattern-based grouping of the sequences (Fig 6). The method is not only suitable for (ordered) time-series data sets, but can also be applied to other types of comparative experiments such as wild-type versus multiple treatments or cross tissue comparison. Grouping DE sequences allows users to quickly view sets of sRNAs that follow the same pattern of expression throughout the experiment.

During our analyses we observed that problematic data sets arise when whole size classes are affected by a condition, causing a high rate of DE for a large proportion of the sRNAs, e.g., RNAi mutants which cause the exclusion of a whole class of sRNAs or virus infections which produce a large set of viral siRNAs in the infected samples (Szittya et al. 2010). To our knowledge no current normalization is able to correct for such experiments, and further approaches will need to be developed to provide an appropriate normalization solution to this kind of data.

In conclusion, we have described a user-friendly pipeline for sRNA DE analysis that allows the evaluation of a variety of techniques to identify the most suitable approach for a given data set. The workbench includes both established approaches and tools that we have specifically developed for sRNA sequence analysis and facilitates a coherent and informed analysis through linking the different aspects into a workflow. The UEA sRNA Workbench and the pipeline design devised for the data analysis may prove to be a valuable resource facilitating the expansion of our knowledge of sRNAs, especially for the study of new or less well characterized classes of sRNAs.

## MATERIALS AND METHODS

To illustrate the use of the pipeline we use a *H. sapiens* data set referred to as “H” data (publicly available on Gene Expression Omnibus [GEO] under accession number GSE47532). This is an experiment on the effects of hypoxic conditions on MCF7 cells (Camps et al. 2014), organized into a time series of four points, each with two biological replicates, Normoxia (N00), Hypoxia at 16 h (H16), Hypoxia at 32 h (H32), and Hypoxia at 48 h (H48). The additional examples presented in the Supplemental Information are based on publicly available *B. terrestris* data (GSE64512) consisting of two samples, with four biological replicates each (Sadd et al. 2015) and a publicly available *A. thaliana* data (GSE35562, GSM1178880 to GSM1178882 for the wild-type and GSM1178883 to GSM1178885 for the Hen1-8 mutant) consisting of two samples, with three biological replicates each (Zhai et al. 2013).

In this section, we describe the methodology and software underpinning the new pipeline; the main workflow diagram is presented in the diagram in Figure 2A.

### Quality checking

The sequencing quality of individual sRNA-seq samples is assessed based on properties such as the positional nucleotide composition (SEQC/MAQC-III Consortium 2014), sequencing depth and the number of unique sequences present in a sample (Rajagopalan et al. 2006). The accuracy of expression replication is evaluated by comparing, qualitatively and quantitatively, the abundances of reads between replicates (Mapleson et al. 2014). The quantitative analysis includes the study of size-class separated distributions of abundances and complexities, defined as the ratio of unique (non-redundant) to total (redundant) reads and the Jaccard similarity index on the top 500 most abundant reads (Jaccard 1901; Mohorianu et al. 2011). The qualitative comparison is conducted through the replicate versus replicate scatter plots and MA plots/Bland–Altman plots (Bland and Altman 1986). We also assess the stability of distributions of fold changes between replicate libraries for each size class presented in log_2_ scale. An appropriate similarity between the compared replicates/samples is indicated by tight distributions, symmetric on 0 log_2_ fold change with no deviations for any particular size classes (Yang et al. 2002; Mohorianu et al. 2011). The percentage of genome-matching reads is calculated for both redundant and non-redundant sequences and across size classes. Selected annotations for which similar checks are performed include miRNAs, other ncRNAs (such as tRNAs, rRNAs, or snoRNAs), protein-coding genes, and repeat/transposable elements depending on available annotation information (Xu et al. 2014; Omidvar et al. 2015).

Abundance distributions of reads in each sample are plotted in a series of boxplots (McCormick et al. 2011; Dillies et al. 2013). However, due to the high proportion of low abundance reads characteristic to sRNA-seq data these distributions for all reads are often uninformative. To counter this, we break the data into abundance ranges of user-defined length (referred to as abundance windows) and assess the comparability of the sample distributions within each window.

### Normalization

The aim of the normalization of the expression levels is to minimize the technical variation between replicates and treatments which is not biologically relevant, e.g., sequencing errors and biases or artifacts from the RNA itself (Sorefan et al. 2012; Raabe et al. 2014) since DE predictions are only considered reliable when the variability between replicates is lower than the differences between the treatments. In the Normalization component of the pipeline, we incorporate several existing methods for normalization (scaling-based, rank-based, and statistical), with additional features, adapted for sRNA data sets. Scaling normalizations, based on the identification of a scaling factor which brings the total number of reads to an a priori fixed total include: the reads per million (RPM)/reads per total (RPT) method (Mortazavi et al. 2008) for which the total abundance of all reads in a sample is considered, upper quartile normalization (Bullard et al. 2010) for which only the reads with abundances in the upper quartile are considered, the trimmed mean of M-values (TMM) (Anders and Huber 2010) and DESeq (Anders and Huber 2010).

Quantile normalization (Bolstad et al. 2003), originally designed for microarray experiments, is also included as an option in the pipeline. This method imposes the same distribution of ranks over all sequences in the data set. We adapted this method to sRNA sequencing data by adding two extra conditions: (i) If, within a sample, two or more reads have the same abundance before normalization, they are assigned the same abundance after normalization which is the average of the normalized abundances. (ii) If a read is not present in the original sample (abundance = 0) then it is assigned an expression level of 0 in its normalized version.

We also include a subsampling-based normalization which is an adapted version of the method described in Li et al. (2012). Our method is based on sampling reads (without replacement) to the minimum library size (for all samples that pass the quality check). It consists of two steps: (i) to ensure that the distribution of abundances are consistent within a sample, the sampling is conducted for decreasing proportions until the sample's distribution has significantly changed or the lowest sample size has been reached; (ii) a subsample of reads with a fixed total is selected repeatedly and, using bootstrapping, the variability of the subsamples is tested. If the variability is low, a random sample (representative for the distribution, i.e., not an outlier) is selected.

### Differential expression call

To identify DE sequences between conditions/treatments, the pipeline includes a confidence interval (CI)-based approach (Lopez-Gomollon et al. 2012; Mohorianu et al. 2013). For each sequence, in each condition, a CI is calculated over replicate expressions using either Chebyshev's intervals calculated from the mean and the standard deviation (Singh et al. 2006) or the minimum and maximum expression levels if only two replicates are available. For a selected comparison between a reference and observed condition, the direction of DE and its amplitude are also calculated. A directional descriptor from the set (up [U], down [D], straight [S]) is assigned to each sequence as follows: S is used if the CIs overlap, U indicates that the observed CI is higher than the reference, and D indicates the opposite result. The issue of performing pairwise comparisons with sample counts greater than two can then be addressed by forming patterns using the (U,D,S) descriptors. This allows sorting and filtering of sequences that result in potentially relevant/interesting expression changes throughout the course of the experiment.

The amplitude of the difference in expression between conditions is considered on proximate extremes (the closest ends of the neighboring CI) of the reference and observed CIs and is only calculated on sequences that have been assigned a U or D descriptor. The amplitude is calculated using the log_2_ offset fold change (LOFC) method previously described in Mohorianu et al. (2011, 2013). The offset prevents low abundance variation from being included in the significant DE distribution. The aim of the offset-approach is to reduce the number of false positives from low abundance sequences and to allow fold change values to be used directly when assessing the relative significance of differentially expressed sequences.

To determine an appropriate offset for a data set, the pipeline can be used to estimate the abundance level around which the majority of noise-related reads lie. Previous studies have observed that low abundance regions/loci have a high strand bias (derived from the reduced number of reads), but loci within the noise-to-signal range have no preferred strand bias (Mohorianu et al. 2011). Based on this observation, the method assigns sRNAs to windows of a set length along the genome reference and the total expression and strand bias is then calculated for each window. For all expression levels, the distribution of strand biases is compared to a random uniform distribution using the Kullback–Leibler (KL) divergence measure (Kullback and Leibler 1951). We define the noise-to-signal threshold (the offset) as the value for which the global minimum of the KL divergence distribution is reached. The distribution is smoothed by a LOESS function (Cleaveland 1979) to prevent expression level outliers from giving a local minimum. Expression levels lower than this threshold tend to have a higher divergence from a uniform strand bias due to a low number of incident reads, and expression levels that are higher than the threshold have an increasing divergence measure due to biologically relevant reads.

## DATA DEPOSITION

The workbench and all the supporting data and tutorials are freely available from http://srna-workbench.cmp.uea.ac.uk. The license is a custom license written for the UEA sRNA Workbench and can be found in the Workbench installation directory or by visiting http://srna-workbench.cmp.uea.ac.uk/wp-content/uploads/2016/11/sRNA-WorkbenchEULA.pdf. There are no restrictions on use other than requiring citations to specific papers when conducting research with the software; specific details can be found on the website.

## SUPPLEMENTAL MATERIAL

Supplemental material is available for this article.

## Supplementary Material

Supplemental Material
