# Supplementary material for: Comprehensive processing of high-throughput small RNA sequencing data including quality checking, normalization, and differential expression analysis using the UEA sRNA Workbench
Source: RNA. 2017 Jun;23(6):823–35. doi: 10.1261/rna.059360.116 (PMC5435855; doi:10.1261/rna.059360.116)
Supplement: Supplemental Material [file supp_23_6_823__index.html]

Comprehensive processing of high-throughput small RNA sequencing data including quality checking, normalization, and differential expression analysis using the UEA sRNA Workbench — Supplemental Material 

# Comprehensive processing of high-throughput small RNA sequencing data including quality checking, normalization, and differential expression analysis using the UEA sRNA Workbench

## Supplemental Material

- Supplemental\_Material.docx
