# Supplementary material for: Comprehensive processing of high-throughput small RNA sequencing data including quality checking, normalization, and differential expression analysis using the UEA sRNA Workbench
Source: RNA. 2017 Jun;23(6):823–35. doi: 10.1261/rna.059360.116 (PMC5435855; doi:10.1261/rna.059360.116)
Supplement: Supplemental Material [file supp_059360.116_Supplemental_Material.docx]

**Supplementary Information 1 – sRNA analysis conducted on *B. terrestris* data, consisting of 2 samples, with 4 biological replicates each.**


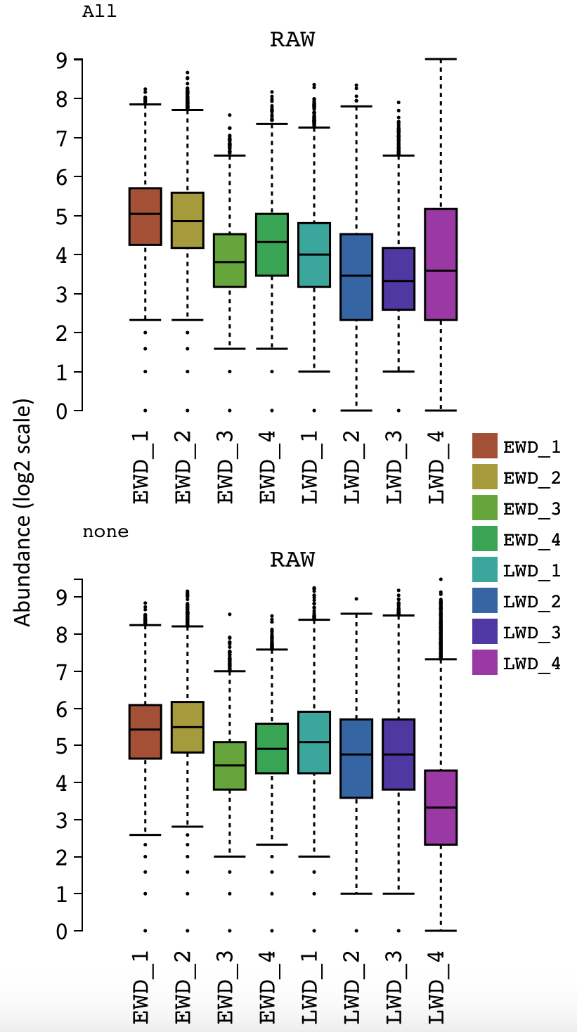


**Figure SI1**. **Distribution of abundances (in log_2_ scale) of *B. terrestris* sRNAs for (a) all reads and (b) reads not matching to the reference genome**. The two samples (EWD and LWD) consist of 4 biological replicates each (numbered 1 to 4). The distributions are represented as standard boxplots, with the box being the inter-quartile range, the horizontal line indicates the median, the whiskers extend to 5% and 95%, respectively, and the outliers are represented with dots. Prior to normalization, the distributions are not aligned, and therefore not comparable.


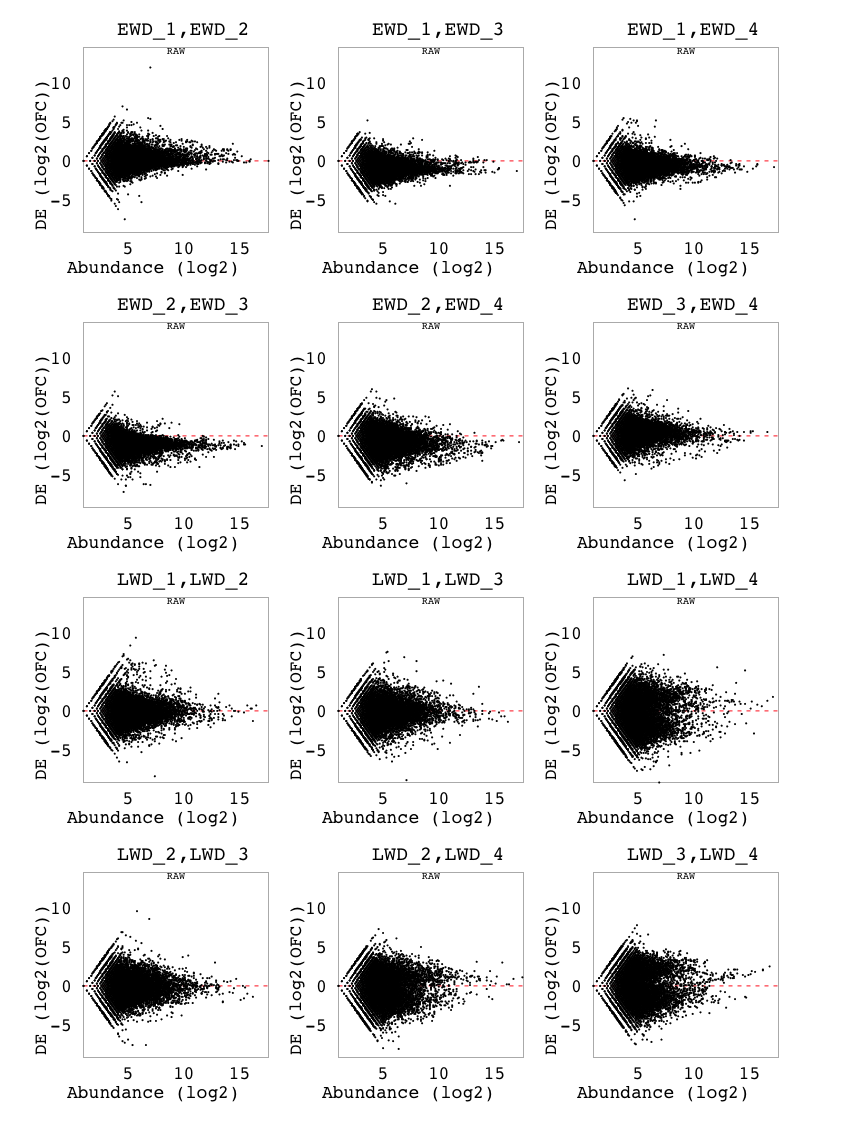


**Figure SI2**. **MA plots for all pairwise comparisons of the *B. terrestris* sRNA data, prior to normalization and prior to genome matching.** On the y-axis we represent the log_2_(OFC), with offset=20; on the x-axis we represent the average abundance (in log_2_ scale) of each sRNAs in the two samples which are compared. A funnel-like distribution indicates a good comparability of the replicates (e.g. EWD1 vs EW2; LWD1 vs LWD3), while a scattered distribution indicates variability in the sRNA population (using a voting approach, the problematic sample can be identified and further scrutinized e.g. the comparisons LWD1 vs LWD4, LWD2 vs LWD4 and LWD3 vs LWD4 indicate that the LWD 4 sample is problematic)


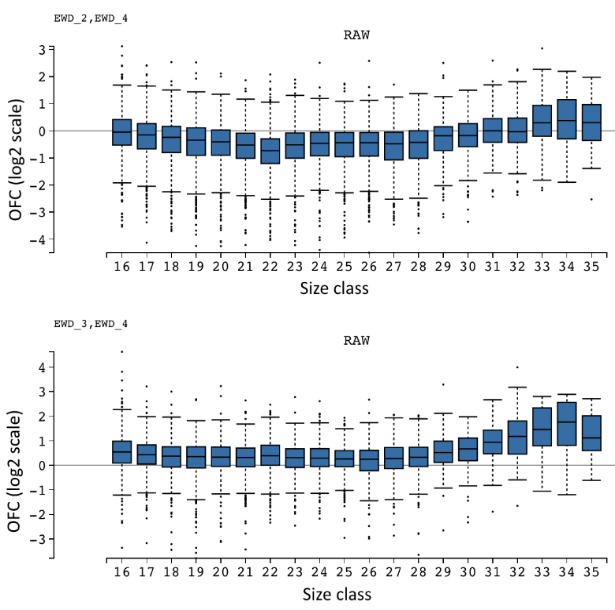

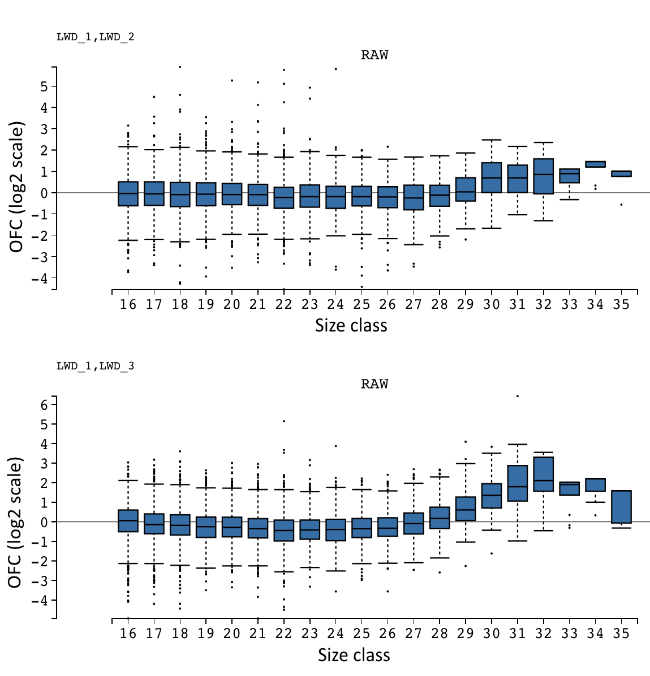

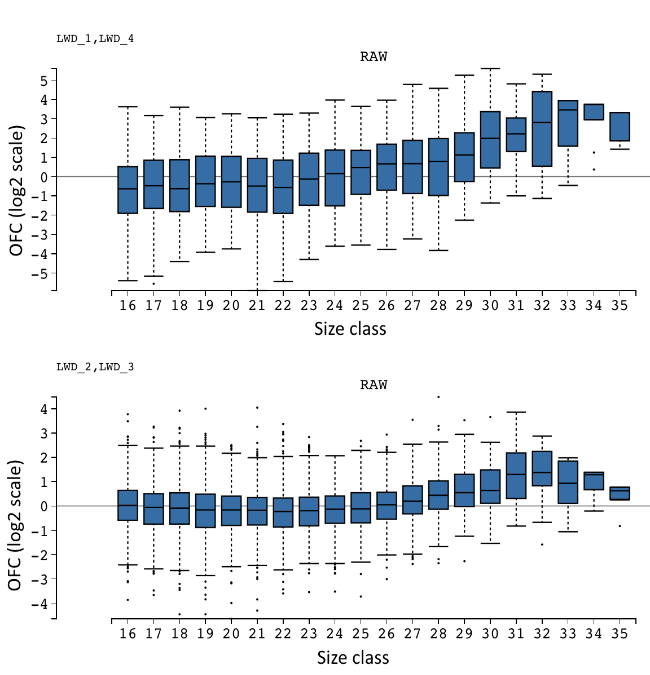

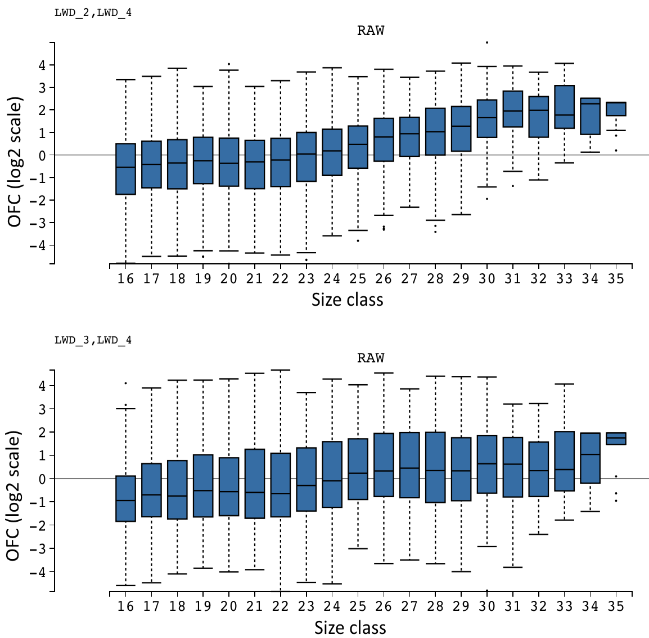

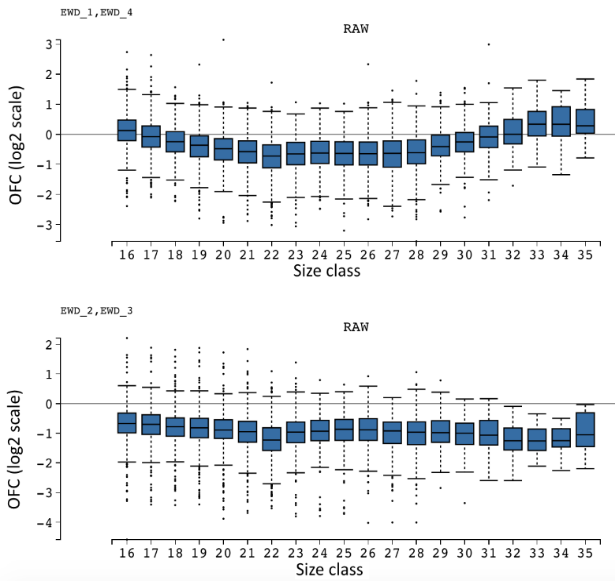

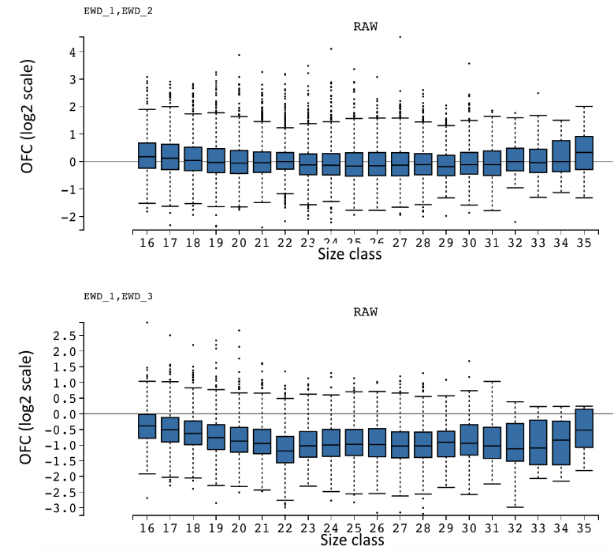


Figure SI3. **Pairwise distributions of differential expression (calculated as an offset fold change, offset = 20), prior to normalization, represented on the y-axis, for each individual size class, represented on the x-axis.** To evaluate the comparability of the biological replicates, using the reads in each size class, we represent the replicate-to-replicate differential expression as standard box-plots. Highly comparable replicates, after the normalization of expression levels, would show narrow distributions (small IQR), centred on the 0 OFC line, which in linear scale, correspond to 1 FC i.e. no differential expression.


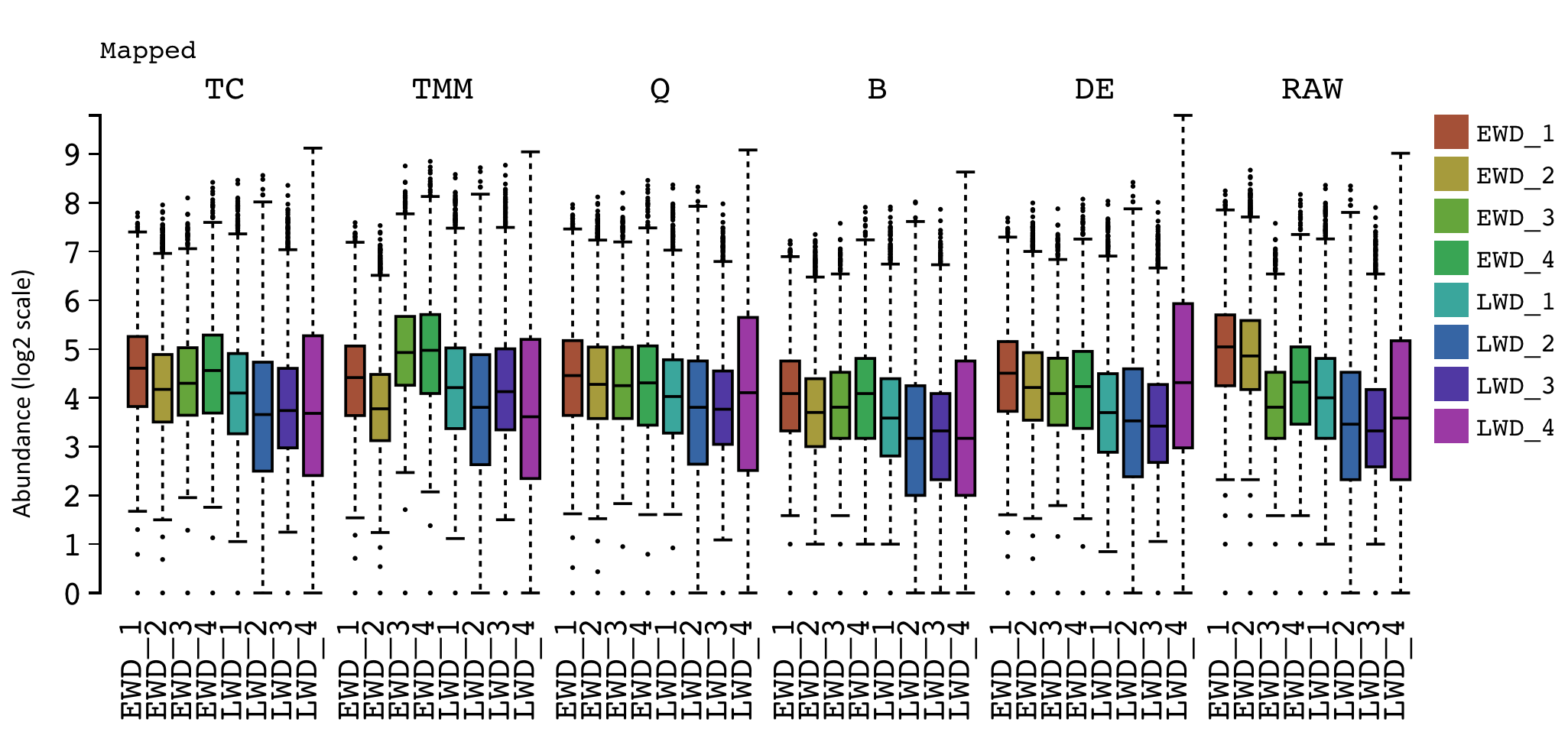


Figure SI4. **Distributions of abundances (in log_2_ scale) for various normalizations (a) TC, total count normalization, conducted on the normalization total of 3.286M (Mortazavi et al. 2008), (b) TMM normalization (Anders et al. 2010)[ref], (c) quantile normalization (Bolstad et al. 2003), (d) subsampling-based normalization (Li et al 2012), (e) DESeq normalization (Love et al 2014), (f) raw data.** After normalization, we expect the distributions of abundances to become comparable. For this particular dataset, the quantile output, the DESeq output and marginally the TC and the subsampling output produce comparable distributions. The TMM approach is not recommended on this dataset. In addition, all normalizations indicate that LWD4 sample is different from the other replicates in the LWD sample (this was indicated by the MA plots, figure SI2).


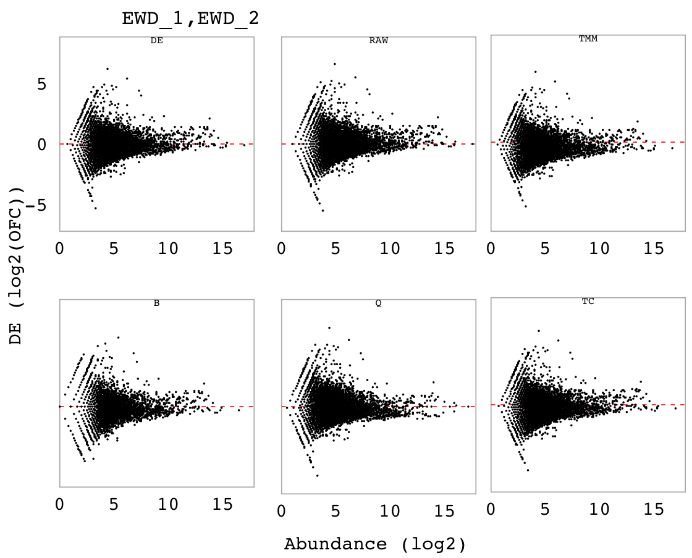

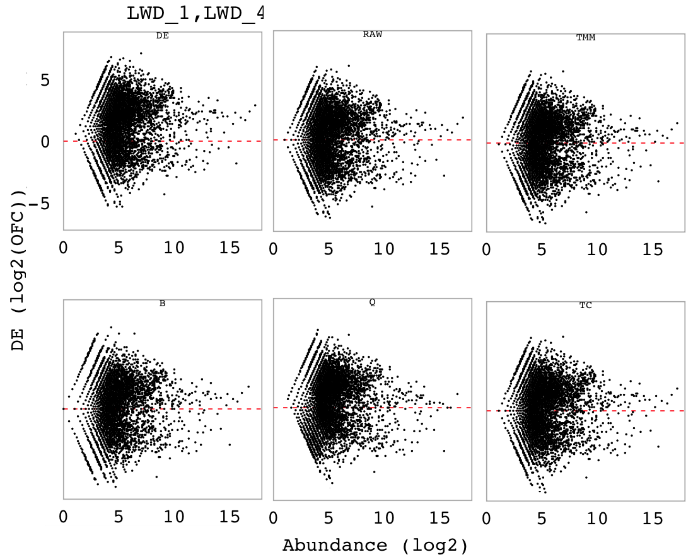


**Figure SI5**. **MA plots for pairwise comparisons of the *B. terrestris* sRNA data (EWD1 vs EWD2 and LWD1 vs LWD4), for all 6 normalization approaches.** On the y-axis we represent the log_2_(OFC), with offset=20; on the x-axis we represent the average abundance (in log_2_ scale) of each sRNAs in the two samples which are compared. A funnel-like distribution indicating a good comparability of the replicates is preserved for the EWD1 vs EW2; the scattered distribution indicating variability in the sRNA population cannot be corrected by any normalization for the LWD1 vs LWD4 comparison, supporting the earlier conclusion that the LWD 4 sample is problematic)

**Supplementary Information 2 – Runtime analysis using 2, 3 or 4 replicates**

To evaluate the variation in runtime corresponding to the size of the dataset, we conducted the analysis of the *B. terrestris* data using 2, 3 or all 4 replicates, for the two available samples, EWD and LWD. The available samples have an average of 4M reads per replicate, and currently could be described as medium datasets (assuming that datasets of approx. 1M reads per replicate are small, and datasets of approx. 20M reads per replicate are large).

In Table SI6 and Figure SI7 we present the runtimes for limited amount of RAM (5GB and 10GB) and for an experiment conducted exclusively in RAM (RAM ONLY). While it is clear that the runtime is linked to the amount of available RAM, it is important to note that the UEA sRNA Workbench uses a disk based architecture i.e. frequently information is streamed to and from the hard disk to prevent runtime exceptions which occur when the maximum available RAM is exceeded. This means that the disk read/write speed is as important as the amount of available RAM.

The desktop computer used for testing the UEA sRNA Workbench has a specification of a 2.6GHz Intel Core i7 processor with 16GB of RAM and uses solid state storage. The server used for these experiments is comprises an Intel 2.67GHz Xeon processor and 200GB of total RAM, however, only has access to magnetic disks, which affects the runtime. Therefore the server tests were conducted in “RAM only” mode foregoing the need to utilise disk (this is part of the design allowing users with high end computers to run the software in “fast mode”).

| RAM | Sample Count | Replicate Count | Runtime (hour:min:sec) |
| --- | --- | --- | --- |
| 5GB | 2 | 2 | 1:34:49 |
| 5GB | 2 | 3 | 4:48:28 |
| 5GB | 2 | 4 | 6:55:50 |
| 10GB | 2 | 2 | 0:56.35 |
| 10GB | 2 | 3 | 3:58:02 |
| 10GB | 2 | 4 | 6:10:51 |
| 100GB (RAM ONLY) | 2 | 2 | 0:28:05 |
| 100GB (RAM ONLY) | 2 | 3 | 0:44.35 |
| 100GB (RAM ONLY) | 2 | 4 | 1:35:56 |

**Table SI6: A series of timed experiments conducted on the *B. terrestris* dataset (GSE64512) using 2, 3 or all 4 available replicates.** The UEA sRNA Workbench allows for limits to be placed on total RAM usage, this in turn affects total runtimes. Here we present this effect by conducting identical runs (QC, Normalisation and Differential Expression) on varying number of replicates from the same input data. We consider any computer capable of assigning 100GB or more RAM to a single task as being in the high end range and amounts of around 10GB in the desktop range.

**Figure SI7**. **Runtime on the B. terrestris data, when 2, 3 or all 4 replicates are used conducted for variable amounts of available RAM (5GB, 10GB or 100GB – RAM ONLY).**

**Supplementary Information 3 – sRNA analysis conducted on *A. thaliana* data**


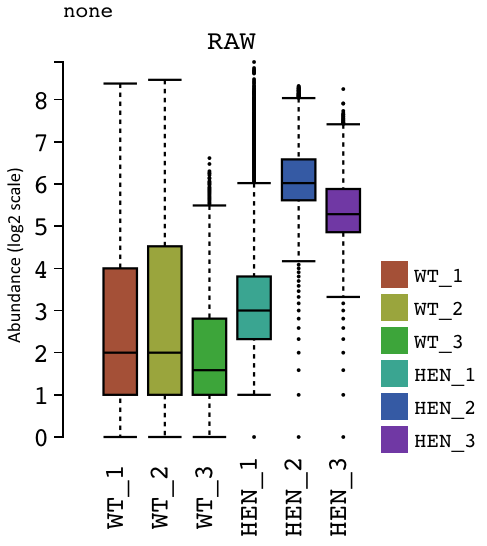


Figure SI8. **Distribution of abundances (in log_2_ scale) of *A. thaliana* sRNAs for all reads.** The two samples (WT and HEN) consist of 3 biological replicates each (numbered 1 to 3). The distributions are represented as standard boxplots, with the box being the inter-quartile range, the horizontal line indicates the median, the whiskers extend to 5% and 95%, respectively, and the outliers are represented with dots. Prior to normalization, the distributions are not aligned, and therefore not comparable.


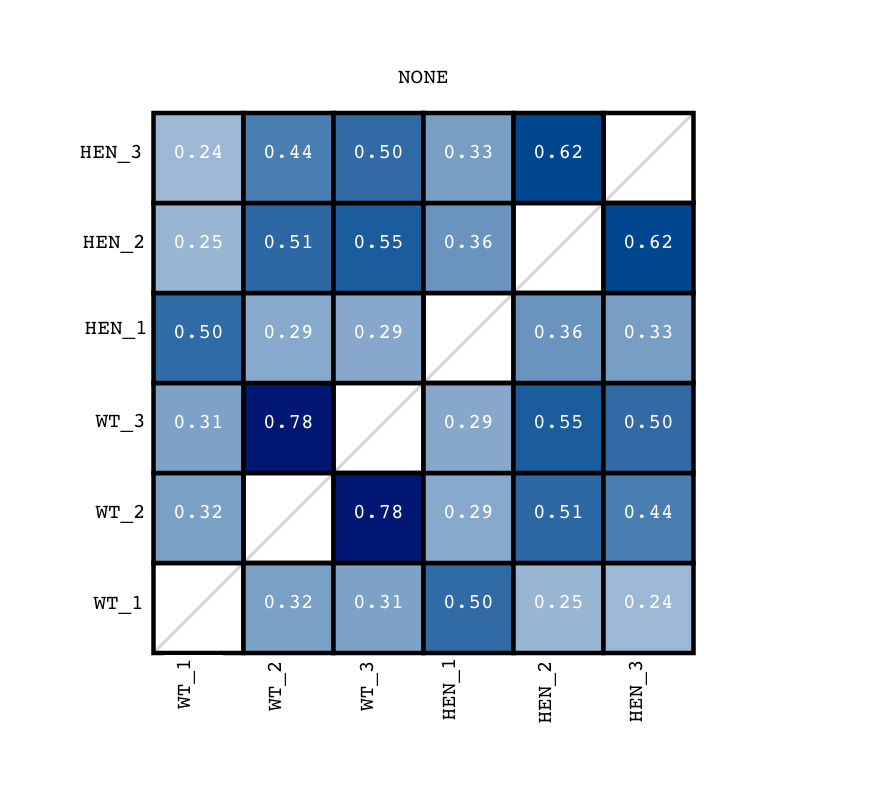


Figure SI9. **Jaccard similarity indexes for the top 500 most abundant *A. thaliana* sRNAs.** The two samples (WT and HEN) consist of 3 biological replicates each (numbered 1 to 3). For a reliable identification of differentially expressed sRNAs, the replicates are expected to be more similar than the treatments. For the WT samples, replicates 2 and 3 have a Jaccard similarity index of 0.78, indicating a high reproducibility; however, replicate 1 has a Jaccard index of 0.31 and 0.32 with the other wt replicates, but 0.5 with the third HEN replicate. Similarly, replicate 1 in the HEN samples shows less similarity with replicates HEN2 and HEN3. This step of the analysis suggest that further investigations are required for the first replicate in WT and HEN samples.


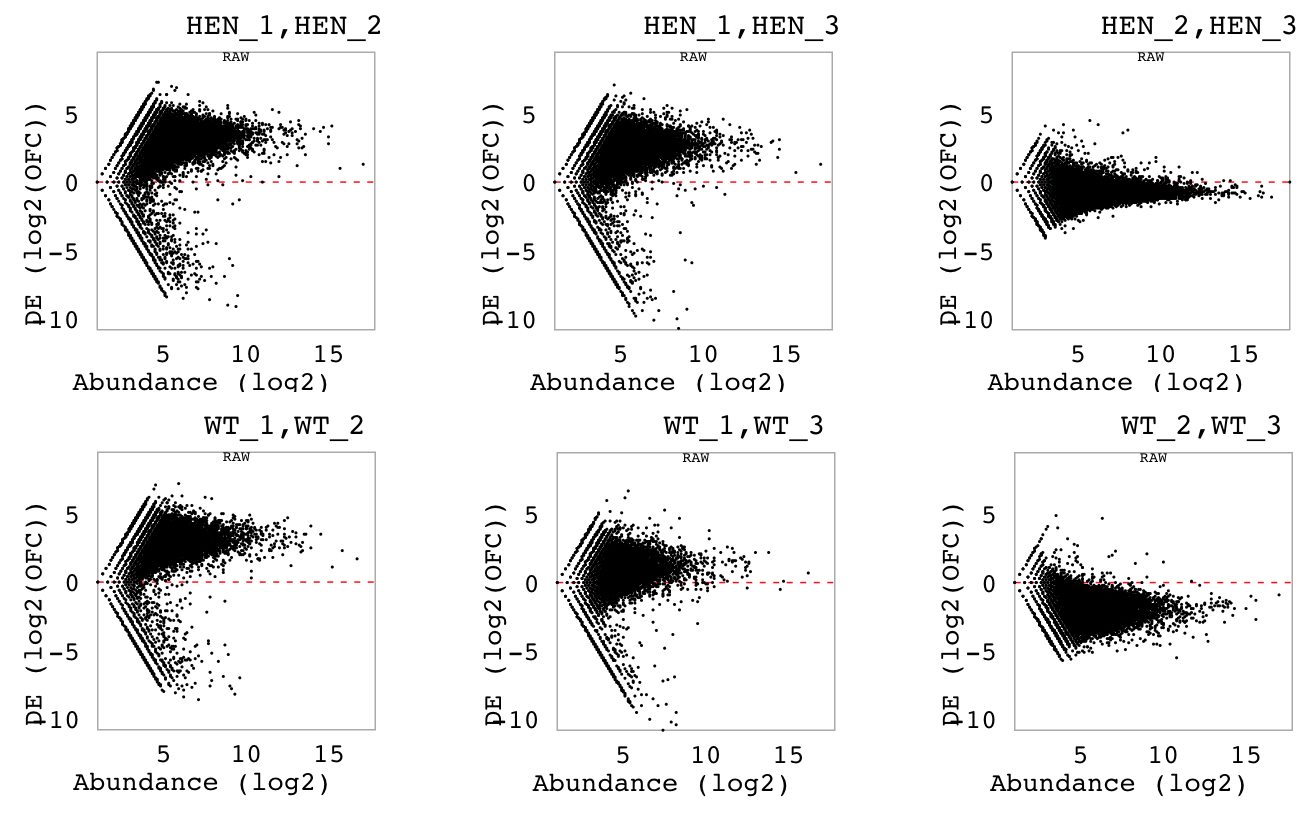


Figure SI10. **MA plots showing the pairwise comparison of replicates, for all reads, prior to normalization, for the WT and HEN samples.** On the y-axis we represent the log_2_(OFC), with offset=20; on the x-axis we represent the average abundance (in log_2_ scale) of each sRNAs in the two samples which are compared. A funnel-like distribution indicating a good comparability of the replicates is observed for the HEN2 and HEN3; the pulled distribution indicating the presence of a whole class of sRNAs in the sRNA population is observed for HEN1 and WT1, respectively.


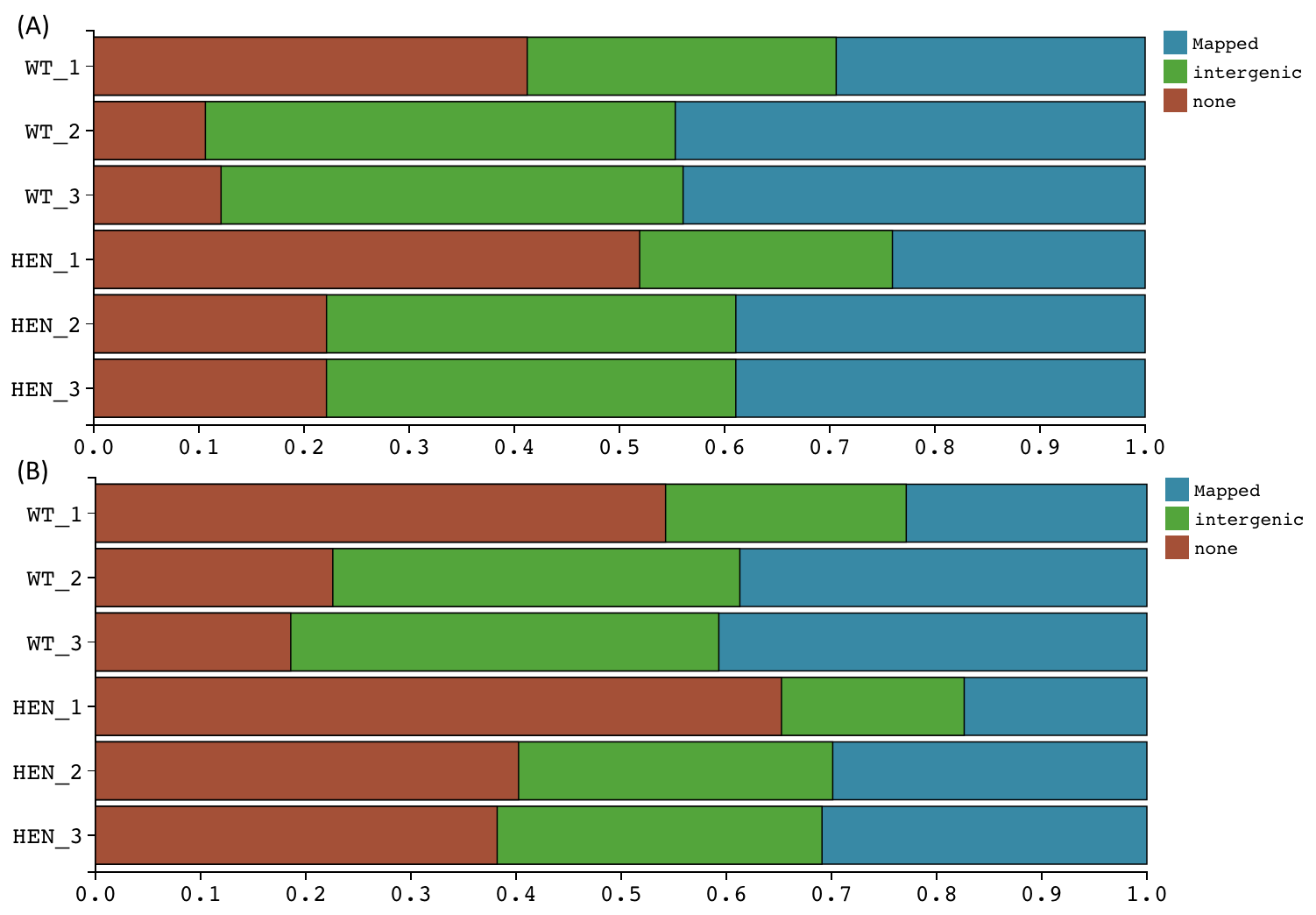


Figure SI11. **Proportions of reads matching to the reference genome (mapped reads), intergenic regions (intergenic reads) and not matching to any known transcriptome (non reads) for the redundant reads (A) and the non-redundant reads (B).** In line with the previous results, WT1 and HEN1 consistently show a higher proportion of “none” reads than the other two replicates.

Figure SI12. **Size class distributions for redundant reads (A), non-redundant reads (B) and the resulting complexities (C).**
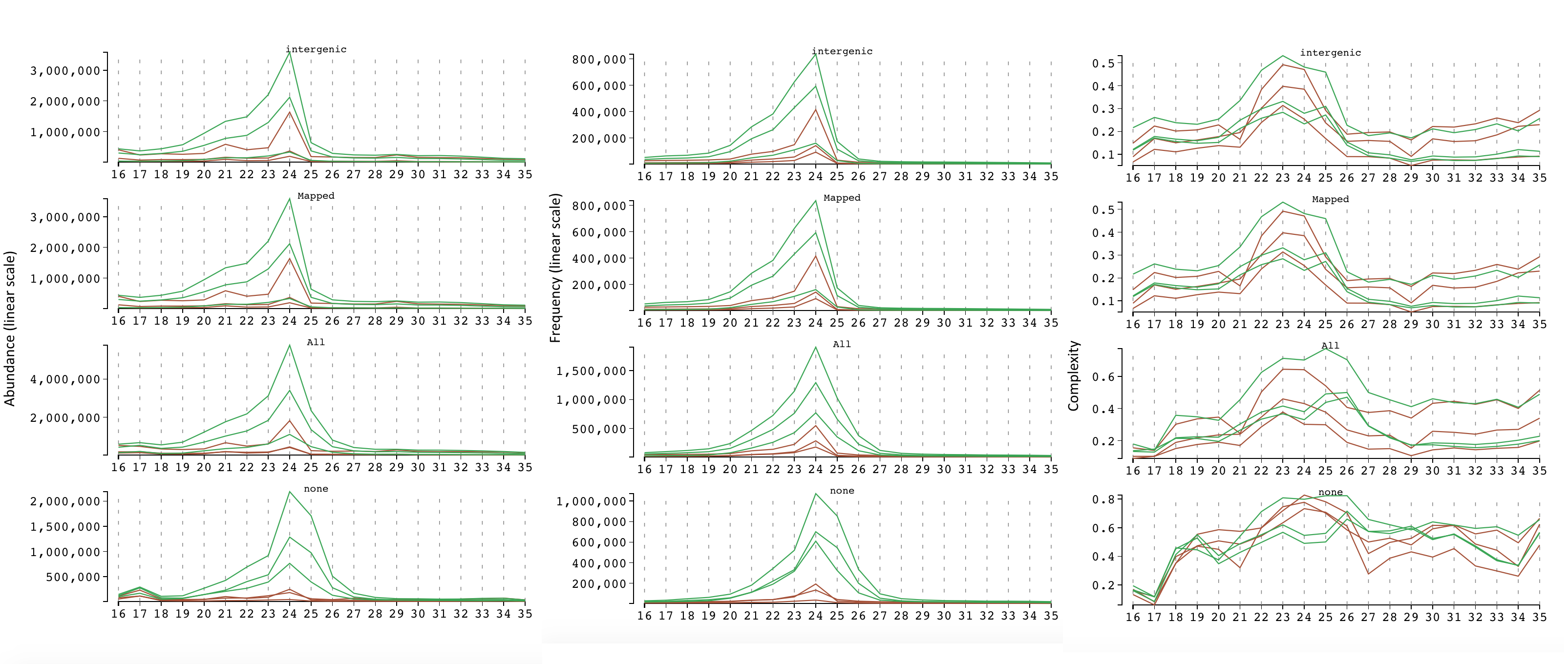
Both the redundant and non-redundant distributions show a peak at 24mers, usually classified as heterochromatin siRNAs. The distributions of complexities show consistently (for mapped reds, intergenic reads and all reads) a lower complexity for the 21mers, mainly miRNAs and a higher complexity for 23mers and 24mers, mainly heterochromatin siRNAs. The complexity distribution for reads not matching to the reference genome does not exhibit a clear pattern; this, coupled with the overall increase in complexity indicates the presence of many diverse, low abundance sRNAs
